# Instrumental Precision in Dental Practice: A Review of Some of the Investigations of Dr. G. V. Black

**Published:** 1901-01

**Authors:** William Leon Ellerbeck

**Affiliations:** Philadelphia


### International Dental Journal.

Vol. XXII. January, 1901. No. 1.

49

### Original Communications.[1]

### INSTRUMENTAL PRECISION IN DENTAL PRACTICE: A REVIEW OF SOME OF THE INVESTIGATIONS OF DR. G. V. BLACK.[2]

BY WILLIAM LEON ELLERBECK, D.D.S., PHILADELPHIA.

In the May number of the *Dental Cosmos* for 1895 there appeared the beginning of an article by Dr. G. V. Black, entitled "An Investigation of the Physical Characteristics of the Human Teeth in Relation to their Diseases and Practical Dental Operations, together with the Physical Characters of Filling-Materials." The article extends through several issues of the *Cosmos*, some important conclusions appearing in the April number for 1896.

It is no exaggeration to say that it is one of the most extensive, one of the most interesting and highly instructive contributions bearing on dental science that has ever been produced, and, as remarked by Dr. Burchard, may properly be mentioned along with the discoveries of Dr. Miller relative to the causative influences of

[1] The editor and publishers are not responsible for the views of authors of papers published in this department, nor for any claim to novelty, or otherwise, that may be made by them. No papers will be received for this department that have appeared in any other journal published in the country.

[2] Read before the Academy of Stomatology, November 26, 1900.

tooth-decay, and of the investigations of Dr. Cryer on the important subject of regional anatomy and its bearing on oral surgery. While, as mentioned, the contribution is highly scientific and decidedly interesting and instructive, it is, however, safe to say that the average dental practitioner has not found time to go very fully into the subject or follow out in detail the whole list of Professor Black's experiments, much less to take up the matter from a practical experimental stand-point, and attempt to verify, or disprove, or improve upon his results.

While it is hardly possible that there is any one here who has been unfortunate enough to have failed to read the article referred to, still there may be some of you who have read but have not seen put into practical operation any of the experiments or tests as performed by Dr. Black. If such there be, it will furnish me the excuse for accepting an invitation to give a partial review of this work, including by way of demonstration a few practical experiments as an argument that his conclusions are not wholly theoretical.

While it is impossible and, in fact, unnecessary at this time to go into the whole detail of his work, I do hope to give you in a general way a review of Dr. Black's experiments, together with some of the important deductions which have a practical bearing on dental practice. In order to acquaint the profession with the exact ground covered in his stupendous work, Dr. Black's article included of necessity much detail, and extended over such a long period of time, it may be that some have failed to properly relate one with the other the several features of his experiments.

First, it appears that his intentions were to determine for careful comparison the exact percentage composition as regards lime salts, organic matter, and water of the different teeth of different individuals of the same and different ages, and of the different teeth of the same individual, noting in every instance the apparent quality of the teeth as adjudged by clinical classification, known as hard, medium, or soft teeth, and also making note of the condition of the teeth at the time of their loss to the patient, whether the pulp was living or dead, whether the teeth had been lost through disease or otherwise, and whether carious or not, the idea throughout being to learn what influence, if any, differences in percentages of lime salts might have in determining the liability of the teeth to assume pathological conditions.

Secondly, but immediately connected with this division, are his experiments determining the hardness of tooth-structure, but more particularly as representing the bulk of the tooth, the elasticity and hardness of the dentine of the different teeth under the different conditions above referred to.

Thirdly, after determining the ability of the teeth under various conditions to withstand crushing stress, there was conducted a series of experiments to determine the force exerted in the closure of the jaws, the object of the experiment, of course, being to learn the amount of stress to which teeth under different circumstances would be subjected, this to be borne in mind in the preparation of cavities for the insertion of fillings, also to determine the strength requirements of the filling-materials to be used.

Fourth, a series of experiments was conducted to determine the effect of dehydrating tooth-structure, noting the difference in degree, if any, of the shrinkage produced in dentine and in enamel, and noting what effects might be produced which would have any influence on our treatment, as regards hot-air applications, in the preparation of teeth for filling.

Fifth, amalgam being extensively employed in tooth repair, an extensive series of experiments was conducted in relation to this material, the effort being to obtain as nearly as possible a product possessing such chemical and physical properties as would insure tooth preservation. Also in this connection were made many experiments to determine exact methods of preparing amalgam alloys, the best metals to be employed in the alloy, and the amount of each. Also, note was taken of the changes brought about by the influence of heat on the cut alloy, and the influence of different proportions of mercury employed to effect amalgamation, and the amount of stress required, and the best pluggers to be used in packing amalgam in order to get the best and uniform results, copper amalgam as a filling-material coming in for its share of attention.

Finally, an extensive series of experiments on gold as a filling-material was conducted, the influence of manipulation and preparation for particular purposes receiving considerable attention.

In regard to the instruments employed by Dr. Black in his experiments I have this to say as to their accuracy: There are four particular instruments used for different purposes in the experiments which register pounds avoirdupois. One is the phagodynamometer, used in determining the force necessary to crush different food-stuffs. Another is the dynamometer, used to determine the crushing stress of blocks of dentine and of amalgam. Another is the mandudynamometer, used to determine the hand-force required in inserting amalgam fillings in order to obtain the best and uniform results; and still another is the tuptodynamometer, used in determining the force of blows required to properly condense gold fillings.

Now, the dynamometer registers on a dial the number of pounds applied on a double bow spring, which, when pressure is applied, moves a rod, which, in turn, moves a cog-wheel, which causes to revolve a hand over the dial-plate which registers the pounds. Some definite way was determined to insure accuracy in the projection of this scale. As to just the method employed I am not aware, but it is plain to see that by applying known weights to the spring and marking the registrations, the divisions could be accurately determined. Now, as to the mandudynamometer, the principle of its construction is somewhat on the principle of a weigh scale, only, instead of the weight register moving up and down, it moves in a plane back and forth. If, now, different weights were applied, and the movement of the arm noted, a scale could be easily projected. Similarly the scale of the tuptodynamometer and phagodynamometer were projected. If a flat-end plugger of known area is arranged on the dynamometer, and a piece of tough paper of definite thickness and texture is placed over the smooth surface of a piece of hard wood, the whole being placed so that the pressure of the point would come directly on the wood and paper, and the plane of the plugger be made parallel with the plane of the paper, it will be found that the same pressure is invariably required to make the plugger just puncture the paper and leave the paper disk buried in the wood so that its outside surface will be in the same plane as the surface of the wood. Now, if the same plugger be placed in each of the other instruments, and the same block of wood and paper be used, it can be shown that absolutely the same pressure as registered on the different instruments will be required to similarly effect the puncture.

My object in citing this example is, of course, to illustrate in a mild way the absolute accuracy of Professor Black's instruments. Throughout the whole range of experiments conducted, the chemical analysis, the methods of determining the specific gravity, and the instrumentation generally, the work has been exceptionally accurate, and we may depend upon the results, which being the case, I will not burden you with the whole details of how they were brought about, but will content myself with recalling to your minds what they are. To recapitulate, I will say that Professor Black's own summary of the scope of the work on the teeth is as follows:

First, the variation in the percentage of lime salts in the human teeth.

Second, the relations of these variations, if any, to the diseases of the teeth, especially caries, but including diseases of the peridental membrane, the supposed loss of lime salts, and softening of the teeth in pregnant women.

Third, the percentage of lime salts to the strength of the dentine and to the strength of the teeth.

Fourth, the force used in ordinary voluntary effort in biting and in masticating, and the relation of this force to the strength of the teeth.

It did not include possible variation in the composition of lime salts, nor did it include variations in the hardness of teeth to cutting instruments. It was found that the variation in percentage of lime salts in all the teeth employed, regardless of condition or from whom extracted, was so very slight as to be practically none at all, being but a gradual increase of about two per cent. in the lengthy period from youth to ripe old age. Of any differences noted, strange to say, the greatest variations were in the different teeth from the same individual, leastwise as compared with the average difference in the teeth of different persons. In the main this unvarying proportion of lime salts was borne out by the specific gravity, which varied but slightly in the different teeth, the greatest difference noted in over a hundred cases being but .097 of a volume. However, such differences were obtained in particular instances as would lead one to believe that the specific gravity does not entirely depend upon the percentage of lime salts. As to the strength or ability of dentine to withstand pressure without fracture or crushing, it also is not directly dependent on the percentage of lime salts, as we shall subsequently see, the condition of the organic matrix seeming to play a more important part.

It was conclusively proved that there was no withdrawal of lime salts from the teeth of women during pregnancy.

It was proved that there is no difference in the density of teeth lost through disease of the peridental membrane.

It was proved that the average tooth affected by caries is by no means deficient in lime salts, nor did such teeth fall short in point of specific gravity.

It was shown that by subjecting a tooth to a temperature of 200° F. for twelve hours, an average shrinkage of about 1.81 per cent. would result, there being a decided difference, however, in the shrinking capacity of dentine and enamel, it being practically *nil* in the latter as compared. This difference it is which causes the checking which occurs when teeth are dehydrated, which fact should be borne in mind when preparing pulpless teeth for filling, since the pain limit would not be a factor in gauging the permissible extent of our hot-air application.

In relation to the hardness and elasticity of dentine, it was shown that two hundred and fifty pounds was the average stress required to crush blocks of dentine .08 of an inch cube, cut from the various teeth from the different individuals of varying ages, and clinically hard or soft, carious or otherwise. As for the elasticity, the blocks were strained or compressed 2.09 per cent. of their thickness under a stress of one hundred pounds, and compressed 4.03 per cent. under a pressure of one hundred and fifty pounds. The elasticity did not depend upon the density of the teeth or upon the percentage of lime salts, yet it apparently varied somewhat according to the condition of the organic matrix, in proof of which it was shown that marked deterioration in strength and elasticity occurs in pulpless teeth which have been left open until perhaps decomposition has to some degree altered the organic structure.

It was shown that the presence or absence of caries is in no way a factor in determining either the hardness or elasticity of tooth-structure, or *vice versa,* the hardness and elasticity in no way influencing caries. The crushing stress varied from two hundred to three hundred pounds, the high stress almost invariably characterizing the teeth of young people.

The prevailing idea of white teeth being soft was found to be an error, an example of such kind resisting a strain until the application of three hundred pounds.

The strength of dentine having lateral support was shown to be very great, standing easily a stress of three hundred and fifty pounds, even the weakest tooth in need of and suitable for filling being able, when properly prepared, to withstand any crushing stress to which it is likely to be subjected.

It was shown that the enamel is decidedly the inferior of dentine in withstanding crushing stress, the average specimen crumbling at from thirty-five to seventy-five pounds. Therefore the great reason for not unnecessarily cutting away dentine from the enamel walls in the preparation of cavities for filling, as the chances for permanency are materially lessened.

The main points of Dr. Black's conclusions are as follows:

The teeth are strongest in youth and early adult age, diminishing somewhat in strength with advancing years.

Teeth that have lost their pulps and have become discolored lose strength in a marked degree, apparently from the deterioration of the organic matrix.

There is no basis for the supposition that the teeth of children under the age of twelve years are too soft to receive metallic fillings, since they are just as hard, just as strong and dense, and, with the exception of an unimportant and inappreciable difference, contain just as much lime salts as the teeth of adult age. There is, then, only one basis for the selection and adaptation of filling-materials to different cases. This is, for one to select the material which he can manipulate so as to make the most perfect filling considering the circumstances, his own skill, durability of material, and the patient's ability to bear its insertion.

The active cause of caries is a thing apart from the teeth themselves, acting on them from without. It is, perhaps, something in the constitution operating through the oral fluids that is the chiefest factor in determining the liability to caries of the teeth of different individuals, the rapidity and extent of caries depending, of course, upon the activity of the exciting cause. From this the logical inference is that caries of the teeth is not dependent upon any condition of the tissues of the teeth, but upon conditions of their environment.

There is no basis for the supposition that calcic inflammation of the peridental membranes or phagedenic pericementitis (so-called pyorrhœa) attacks persons who have dense teeth in preference to those whose teeth are less dense.

There is no basis for the treatment of pregnant women medicinally with the view of furnishing lime salts to prevent the softening of their teeth, or with the view of producing better calcified teeth in their offspring.

So much for the lime salts, the organic matter, the elasticity, the density, and the hardness of different teeth. Now, as to the stress to which they, and fillings, will likely be subjected. In the experiments conducted to determine the force exerted in the closure of the jaws it was shown that the physical strength of the individual was not the chief deciding factor. Rather the condition of the pericemental membrane was the more potent one, for in biting down on the gnathodynamometer (the instrument used in determining the strength of bite) one does not cease in the closure of his jaws because of lack of strength, but rather because of the pain occasioned. The pounds pressure necessary to produce pain varies greatly with different individuals, those habituated to the mastication of hard food having accustomed the peridental membranes of their teeth to stand more pressure than those whose diet consists chiefly of soups and semi-solid foods. The value of tooth exercise is therefore apparent. The variation in bite of fifty individuals was from sixty to two hundred and seventy pounds, the average being one hundred and fifty pounds. But we cannot take into consideration the average bite, though it may represent that which will be most often met, but we must be prepared to meet the extreme stress to which the teeth and fillings are likely to be subjected. Even so, as above stated, we find that even the weakest teeth, suitable for filling, are amply strong to withstand the stress providing they are properly prepared, which latter feature decides largely the success of the filling-material they are designed to receive, the other factors, of course, being the preparation and manipulation of the material.

It was shown that many fillings inserted by the average practitioner are not at all adapted to withstand the impact occasioned in the mastication of certain hard food-stuffs. Other fillings properly inserted into properly prepared cavities are well able to withstand the strain. The consideration of this leads us to a very important part of this very important work. While Professor Black has taken up amalgam for his first consideration, we will take the privilege of altering the arrangement somewhat, and first briefly call attention to some of the physical properties of gold, the attributes of which metal as a filling-material entitle it, however, to a more careful and extensive consideration than I can at this time pretend to give.

GOLD.

The aim in view in making the experiments with gold was to establish the correlation that exists between the strength of fillings made with this material, the teeth in which they are placed, and the stress brought upon them by the impact occasioned in the crushing of hard food-stuffs, and the occasional biting of hard particles during such process, and incidentally to establish an exact way of determining the efficiency or inefficiency of the different methods of preparation and manipulation of gold, that we may in our operations be governed accordingly.

Experimentation showed that the maximum density of gold very closely approximates 19.4, and this was taken as a basis of comparison. Cast gold of about this density—19.3—in cubes of one-tenth inch section when subjected to a pressure of two hundred pounds shortened 4.12 per cent., and under three hundred pounds the average shortening was 11.21 per cent. The least shortening noticed in any of the specimens of cast gold under two hundred pounds pressure was 2.41 per cent., 9.95 per cent. at three hundred pounds, and 14.43 per cent. at three hundred and fifty pounds, all of which give a good idea of the behavior of cast gold under pressure. As to the effect of hammering, such as would occur during the ordinary process of filling with mallet blows, experiment showed that after a bar had been thoroughly hammered, the average shortening under a stress of two hundred pounds was only .48 per cent., less than one-half of one per cent., as compared with 4.12 per cent. in the case of the cast gold. The average shortening under three hundred pounds was 1.98 per cent., as compared with 11.21 per cent. in the cast but unhammered bar. None of the cubes showed any shortening under a pressure of one hundred and fifty pounds (a point to be remembered), while in the case of the cast gold the minimum shortening noticed in any specimen under this pressure was 1.04 per cent. From this we may judge of the value of rigid hammering of gold fillings exposed to wear compared with the process of inserting such fillings by hand-pressure. As to density, it was found that hammering is not the deciding factor; rather the avoidance of making air-spaces by bridging the pieces of gold is the important point; therefore, in the insertion of fillings not exposed to wear, hand-pressure will answer, since it is possible to obtain adaptation and density in this way, though this method in most instances possesses no advantages over combined burnishing and malleting, and has the disadvantage of being less rigid.

Fillings made by different operators by their different methods of manipulation and with the different preparations of gold they usually employ were tested. The density of each filling—how nearly it approached the standard 19.4—and the different degrees of resistance each had to impact or crushing stress was noted and the results carefully tabulated for comparison, so that conclusions might be drawn as to the value of the different preparations of gold and the methods of insertion for given cases. I do not propose giving the whole table of the comparisons of the various fillings made by different operators, but we may consider one or two which have a bearing on this difference in hand-pressure and mallet-force.

Experiment No. 1. Gold-foil No. 4. Mallet-force as in ordinary method of filling teeth not exposed to great wear. The density obtained was 17.4, and the filling shortened 1.3 per cent. under two hundred pounds pressure, whereas a filling inserted by hand-pressure—White's cylinders unannealed—gave a density of only 14.6, and shortened 31.1 per cent. under pressure. These different fillings were, however, made by different operators, and we will have to make allowances in the matter of a difference in manipulation, though a comparison of the fillings made by hand-pressure and mallet-force shows a great difference in point of strength.

A study of the table shows that the fillings made of non-cohesive gold-foil are much less dense than those made of cohesive. The greatest force that could be employed with comfort to a patient produced with non-cohesive gold a density of only 16. It was found, too, that such fillings go to pieces under very light stress. In relation to lining cavities with a very small amount of non-cohesive gold, and finishing with cohesive, it was found that the strength of the filling in such instances was not materially lessened. When, however, a considerable amount of non-cohesive gold is employed, and a comparatively thin veneering of cohesive used to finish, the strength is notably diminished, though the density is not lessened. The value of this observation to us is that, in the filling of proximal cavities where we wish to place soft gold at the cervix for better adaptation, we must be careful to use only a very limited amount, lest we materially weaken a filling which is exposed to much stress. It has been found, however, in relation to fillings made entirely of non-cohesive gold-foil, when surrounded by four walls, that even though they may press down in the cavity under stress, they adhere to the margin perfectly; when, however, the bulk of filling is soft gold, and a veneer of hard gold is added, this property of readaptation is lost, and failure results; therefore, when a filling is to be largely of non-cohesive gold, it is best to finish with the same. This is a practical point in the filling of occlusal cavities in the teeth of children, where the limit of endurable force for insertion would only make a weak filling of cohesive gold. It is better in such cases to use the non-cohesive variety. Even though no more density is attained, the ability of the material to hug the margins in the event of its condensing upon itself should give it preference.

In reviewing the table given by Dr. Black, it may be noticed that in many instances the densities of the fillings were below 16, and the resistance to crushing stress was very low, some shortening as much as from ten to thirty-two per cent,—too much, in fact, for such fillings to be of service in occluso-proximal cavities much exposed to mastication, the force of which, as will be remembered, is in many instances very great, easily sufficient to cause such a condensation of many of the fillings as to drive them down into the cavities away from the marginal edges, the resultant evils of which are easily apparent.

The experiments show that we should provide for a stress of at least one hundred and fifty pounds, and often for a greater stress. This strength can only be gotten by using cohesive gold, and condensing it well by the application of sharp, hard blows, taking care, meanwhile, to secure sufficient density and adaptation to guard against a leak and consequent decay. A great many failing fillings show that the cavity has not been properly prepared,—the tooth not cut out sufficiently to make a self-cleansing filling, and the cervix usually not properly cut so as to insure a seat for the fillings with area sufficient to support the stress applied on the occlusal portion in mastication. The method, then, of cutting away a part of the occlusal portion of the tooth, forming a dovetail to insure anchorage, and incidentally to bear a portion of the stress of mastication, together with the preparation of the cervix so as to form a perfect seat to withstand the major portion of the stress, at once approaches the ideal in cavity preparation. The anchorage secured by the dovetail groove in the crown portion is sufficient, with a broad seat at the cervix, to keep the filling from tipping. The absurdity of the method of some operators of cutting small grooves and pits into the lateral walls of proximal cavities, with a view of obtaining additional strength, is at once apparent when we consider that it must necessarily weaken the walls some, and the small spurs of gold ordinarily thus produced at the outside would not support a strain of more than fourteen pounds, totally inadequate to insure the filling from tipping away from the walls when much stress is brought to bear, and we have seen that one hundred and fifty pounds is the average; hence the many failures noticed in fillings thus prepared, they being soon forced away from the walls, and the leak produced permitting bacterial invasion and subsequent tooth-decay. It appears that the condition of the pericemental membrane is at times the most serious handicap to the insertion of gold fillings with the force that should be used in order to get the best results in occluso-proximal cavities. The pericemental membrane is very often so sensitive to pressure as to make the force required to produce hardness unbearable, though the teeth are in themselves abundantly strong to withstand this required force.

When such a condition arises it is often occasioned through sensitive dentine, the tooth perhaps having had a cavity for some time, and, being favored during mastication, the peridental membrane has become sensitive from disuse. In such instances Dr. Black recommends that the teeth be filled temporarily with some plastic for relief of sensitiveness, and the patient be directed to bring them into full use. The sensitiveness of the pericemental membrane, under such treatment, will in a few weeks or months pass away, and the malleting may then be borne with ease. This is a practical point worth noting, and would, it seems, in itself warrant our recommending to our patients and the public generally the use of Zweiback, well-rusked crusts of bread, Bent's water biscuits, and such class of foods, they being indispensable for proper tooth exercise, to say nothing of their value as food-stuffs. It would seem that thus strengthening the pericemental membrane would not only make filling operations possible, but render the membrane less liable to disease. The prehistoric races suffered little from pyorrhœa, and whilst their better constitutional condition may have been the chief reason, still the fact that they lived largely on natural food-stuffs requiring great crushing stress insured the pericemental membrane being strong, and this may in part account for their immunity.

Dr. Black found that fillings of a density of 15 and below were sufficiently porous to absorb animal matter, since they contain twenty per cent. of air-spaces. The tests of many gold fillings made by different operators show that the average dentist does not make his fillings dense enough.

The natural deduction from his experiments in relation to density and rigidity is that to make a *dense* filling the gold does not necessarily require heavy malleting, but it does require that it be used in thin pieces, carefully and evenly laid, and that the malleting be complete over the surface of each piece.

Where a *hard, rigid* filling is required, heavy malleting of the gold is necessary, care being taken in the laying of the gold if great density is also required.

As to the size of plugger-points to be used, I would say that Dr. Black has invented an instrument, the tuptodynamometer previously mentioned, which is designed to give us a clearer insight as to the effectiveness of different-sized plugger-points and the force of blows best adapted to the requirements of different fillings. The scope of the work done with the aid of this instrument has not as yet been published, and I do not feel at liberty to speak of many of the interesting facts brought to light by its employment. We may, however, look forward with pleasure to the publication of these experiments, since they throw much light on this important question.

### AMALGAM.

The complete review of Dr. Black's experiments with amalgam would take so much time that I must forego largely their consideration for the present. However, I feel disposed to say a word or two as to certain results obtained.

The discovery of the property of flow in amalgams—that property of change of mass form from molecular motion under stress—seems at once to explain the cause of failure in so many amalgam fillings, the pressure of mastication causing such change in form at times as to drive the filling away from the tooth wall.

As proving the worthlessness of gold and platinum as modifying metals in amalgam alloy, it was shown that they markedly increase flow and lessen rigidity, and are therefore positively detrimental, their only possible virtue being to improve color.

Zinc likewise should not be used, even small amounts imparting a slow but continual expansion.

From the tests made it appears that copper is the only metal which can with impunity be used as a modifying element, since it imparts additional rigidity and lessens flow.

It was proved that silver is the metal which tends to produce expansion in an amalgam, that tin is the element which tends to produce contraction, and that the production of an amalgam that will neither shrink nor expand depends primarily upon a careful apportionment of these metals in the alloy, so that the shrinking produced by the one will be overcome by the expansion of the other.

The effect of annealing, so-called aging, whereby some change takes place in the molecular construction of the alloy, is to lessen the expansion of the alloys which expand, and increase the shrinkage of the shrinking alloys.

An alloy which when fresh cut shows neither shrinkage nor expansion will contract perceptibly after the lapse of time, due to a slow process of annealing.

To produce a non-shrinking alloy which will not change by process of time,—a temperature change,—it is necessary to use silver sufficient to compensate for the shrinkage produced in the process of artificial aging or annealing. Amalgam made with such an alloy will not change.

The alloy which showed the least change when fresh cut was of the formula, silver 65, tin 35, which curiously corresponds closely to the percentage of the metals were they combined chemically as $Ag_2Sn$, in which 64 silver and 36 tin practically represent the composition.

The alloy which showed the least change of form after annealing was found to be 72.5 per cent. of silver and 27.5 per cent. of tin, which curiously represents very closely the percentage of the metals taken as a compound $Ag_3Sn$, which would be 73.3 per cent. silver and 26.7 per cent. tin.

Modifications of these two basal alloys by the addition of five per cent. of copper is permissible, and often advisable, since greater rigidity and less flow is produced, its tendency to discoloration being the only objectionable feature to its employment.

Gold, platinum, zinc, bismuth, cadmium, and aluminum are all objectionable because of imparting undesirable properties, the objection to aluminum being the enormous expansion which it causes.

In concluding this brief consideration of amalgams, I will say that the crushing stress of blocks of a desirable formula, .085 inch cube, shows a resistance of from three hundred and fifty to four hundred pounds, which is amply sufficient, and, taken with unchanging form, makes this material very valuable for stopping carious cavities in teeth not exposed to view.

### INSTRUMENTS OF PRECISION.

Dentists as a class have shown wonderful and exceptional genius in the invention and perfection of mechanical instruments and appliances and their adaptation to various features of their work. On the other hand, they have given too little attention to the invention and perfection of instruments of precision with which to determine effectiveness of methods, and to test wherever possible the value of so-called clinical evidences, and show when they are features of fancy and when matters of fact.

A century or two ago there was no accurate means of determining the changes of bodily heat and the variations of the pulse-beat, both of which, to all seeming evidence, did change under different circumstances. Much difference of opinion prevailed, and arguments were frequent, and of course unsettleable, until Galileo, who, besides inventing the compass to guide us with unerring accuracy in the matter of direction, invented early in the sixteenth century the first crude thermometer which gave the first accurate means of heat determination. Almost simultaneously he suggested the oscillation of the pendulum as a means with which to test the frequency of the pulse-beat. Both instruments ultimately put a quietus on the dissension caused by the difference of opinion regarding these two physiological phenomena, since they gave some accurate and indisputable means of determination. The perfections of these crude suggestions of Galileo give us to-day the accurate vacuum thermometer and the wonderful and valuable instrument of precision known as the watch.

From Galileo down the different opinions regarding physiological phenomena, opinions often widely at variance, have been settled only after the invention of some instrument of precision, until to-day over five hundred such important appliances are in daily use, and each year sees valuable additions.

Particularly from a dental stand-point Dr. Black's inventions are steps in the right direction, and, like many predecessor instruments of precision which have upset men's opinions, they are not cordially greeted by many whose ideas are at variance with the evidence the employment of these instruments brings forth. Why have we boldly stated that gold and platinum impart additional edge strength in amalgams, when, as a matter of fact, so-called edge strength has been proved to be a misnomer, and, so far as its being analogous to crushing stress is concerned, both metals decrease this property? Why have we said that teeth vary greatly in composition as regards calcium salts, when this has been proved to be not true? Why have we said that teeth are not so strong in youth as in adult age, when, if anything, they are stronger? Why have we said that certain diseases of the pericemental membrane attack most frequently dense teeth, when, with an inappreciable difference, they vary not in density? Why will some of us persist in the exclusive use of hand-pressure to build up with gold corners which will be much exposed to wear, when great rigidity cannot be thus obtained? Why do we answer, and present as proof, that our clinical observations have shown that some fillings thus inserted have been very enduring, when we have had no accurate means of determining the stress to which they have been subjected? and that while one or more such cases may have come under our observation, perhaps even in the mouth of some apparently strong and vigorous man, how do we know that it has not been through some strange fatality of circumstance that they have lasted,—perhaps because of having been subjected to but little strain?—for the bite varies greatly in different individuals, and strength of bite is not gauged by the strength of the individual. The answer to these questions is, *that we have based our belief largely or wholly on clinical observation.* Now, clinical observation is not without great value, and could not and should not be dispensed with, for by demanding exact comparisons and scientific explanations it creates the necessity for accurate instrumentation and experimentation; but, after all, it is liable to great error, as the experience of history has shown. If, now, with the employment of such instruments, the evidence produced is at variance with our opinions formed from clinical observation, why cling to them in the light of positive but contrary instrumental evidence?

To illustrate my meaning, I will say that there has been great variance in matters of opinion concerning coagulants. Should we not, in the light of a preponderance of demonstrable evidence, shown by the practical experiments of Drs. Kirk and Truman, accept as true that coagulants are not self-limiting?

Dr. Black's great work goes to show that, since teeth are practically all alike as regards composition and physical characteristics, other than faulty developmental construction such as pits and grooves, they present no special predisposition to caries, the active cause of which he believes to be not a condition of the teeth themselves, but a circumstance of their environment.

Let us not be in too great haste to laugh at this supposition. In 1835 the opinion was first advanced that caries of teeth was caused by development of acid through fermentation at the exact spot of the carious process, but this was not proved until some fifty years later, when Miller, of Berlin, proved the truth of this supposition and demonstrated the nature of the process.

Now, as to the primary exciting cause. Dr. Black has shown the tissues of the teeth to be universally the same, with no special predisposition to caries, and argues the possibility of some constitutional agent operating through the oral fluids exerting the primary causative influence. Careful experimentation by the bacteriologist and chemist may yet prove the truth of his conclusion, in which event, having led up to the suspicion, the proof, besides being of great use in its bearing on dental prophylaxis, will be a further testimonial to the value of accurate methods of scientific research involving the use of instruments of precision.

